# Modification of the equine gastrointestinal microbiota by Jerusalem artichoke meal supplementation

**DOI:** 10.1371/journal.pone.0220553

**Published:** 2019-08-08

**Authors:** M. Glatter, K. Borewicz, B. van den Bogert, M. Wensch-Dorendorf, M. Bochnia, J. M. Greef, M. Bachmann, H. Smidt, G. Breves, A. Zeyner

**Affiliations:** 1 Institute of Agricultural and Nutritional Sciences, Group Animal Nutrition, Martin Luther University Halle-Wittenberg, Halle (Saale), Germany; 2 Laboratory of Microbiology, Wageningen University & Research, Wageningen, The Netherlands; 3 Institute of Agricultural and Nutritional Sciences, Biometrics and Informatics in Agriculture Group, Martin Luther University Halle-Wittenberg, Halle (Saale), Germany; 4 Julius Kuehn Institute, Federal Research Center for Cultivated Plants, Crop and Soil Science, Braunschweig, Germany; 5 Institute for Physiology and Cell Biology, University of Veterinary Medicine, Foundation, Hannover, Germany; Washington State University - Spokane, UNITED STATES

## Abstract

The objective of this study was to investigate the impact of natural prebiotic active compounds on the microbial composition in different regions of the equine gastrointestinal tract. Twelve adult horses (body weight [bwt] 534 ± 64.5 kg; age 14 ± 7.5 years) were randomly divided into two feeding groups. Six horses received a basal diet consisting of 1.5 kg hay/100 kg bwt x d^-1^ and oat grains equal to 1.19 g starch/kg bwt x d^-1^, supplemented with Jerusalem artichoke meal providing prebiotic fructooligosaccharides + inulin in a quantity of 0.15 g/kg bwt x d^-1^. The remaining horses received a placebo added to the basal diet. The horses were fed for 21 d and euthanized at the end of the feeding period. Digesta samples from different parts of the gastrointestinal tract were taken, DNA extracted and the V1-V2 region of the 16S rRNA gene amplified. Supplementation with the prebiotic increased the relative abundance of *Lactobacillus* (*P* < 0.05), with a concurrent reduction of the relative abundance of *Streptococcus* mainly in the stomach (*P* < 0.05). In the hindgut, the supplemental prebiotic also increased the relative abundance of *Lactobacillus* but further reduced the relative abundance of fibrolytic bacteria, specifically the unclassified members of the families *Lachnospiraceae* (*P* < 0.05) and *Ruminococcaceae*. The relative abundance of the genus *Ruminococcus* increased solely in the caecum and colon transversum. Overall, the addition of the prebiotic significantly increased the diversity in nearly all parts of the gastrointestinal tract (*P* < 0.05). The feeding of this natural prebiotic compound to horses had an impact on the microbial community in the entire gastrointestinal tract. Furthermore, the effect on the bacterial community in the foregut (especially the stomach) was more pronounced in comparison to the effect in the hindgut. Therefore, the impact on stomach health should be carefully considered.

## Introduction

The gastrointestinal tract (GIT) of horses is a large and complex ecosystem containing a broad range of different microorganisms [1, 2]. Particularly in the large intestine, horses harbor a specialized microbial community (e.g. *Ruminococcus* spp., *Streptococcus* spp., *Lactobacillus* spp., and *Enterococcus* spp.) which is responsible for fermentation and the provision of fermentative products, e.g. short chain fatty acids (SCFAs) [3]. The stomach also harbors a specific mucosal, as well as a luminal microbial community mainly consisting of *Firmicutes* (e.g., *Lactobacillus* spp., 10^8^−10^9^ CFU/mL), *Bacteroidetes* (e.g. *Prevotella* spp.) and *Proteobacteria* (e.g., *Actinobacillus* spp.) [1, 4, 5]. The microbiota of the small intestine (duodenum, jejunum and ileum) is inhabited by microbial groups (10^6^−10^7^ CFU/mL) [1], which are known for their proteolytic activity [6], mainly species that belong to classes *Bacilli*, *Clostridia* and *Gammaproteobacteria* [7].

The abundance and large diversity of microorganisms in the GIT is essential for efficient fermentation, particularly of nutrients not degradable by endogenous enzymes. Because of its structural complexity and size, the equine digestive tract is prone to disturbances caused by biotic and/or abiotic stressors [8], which ultimately may lead to the development of gastrointestinal-derived diseases (e.g., colic or laminitis) [9, 10, 11]. Prebiotics can help to stabilize the predominantly autochthonous large intestine microbiota by providing substrates for their metabolism and therefore might counteract the development of gastrointestinal-derived diseases [12]. In the nutrition of horses, inulin-type fructans, such as fructo-oligosaccharides (FOS), or inulin itself, are predominantly used as prebiotic substances [13]. The impact on the animal depends mostly on the level of intake of the prebiotic compound and can have either negative or health-promoting effects [14, 15, 16, 17]. Fecal samples are used in most studies to assess the impact of prebiotics on the large intestine microbiota. Because of the need for invasive sampling and/or sacrifice, other parts of the equine GIT are not routinely taken into consideration, and hence, the overall impact on the digestive tract is often not described. Furthermore, literature [18, 19] showed that fecal samples are inadequate to draw conclusions about the microbial composition in the proximal parts of the equine digestive tract. Hitherto, some authors speculated from in vivo or ex vivo results that the fermentation of inulin-type fructans, as well as inulin itself, begins in the foregut [20, 21] and particularly the stomach [21], which might cause negative effects concerning stomach health. In vitro studies support the suggestion that the digestion of fructans in particular (in terms of acid hydrolysis) might not occur in the large intestine alone (as previously assumed) [22, 23, 24]. Consequently, we hypothesized that i) the feeding of FOS + inulin as active prebiotic ingredients originating from Jerusalem artichoke meal has an impact on the microbiota of the entire GIT and that ii) prebiotic compounds act as possible substrates for the microbiota in the stomach. The objective of the study was to evaluate the impact of supplementation with prebiotic FOS + inulin derived from Jerusalem artichoke meal to a hay-based diet on the microbial composition in different regions of the equine digestive tract.

## Material and methods

The Animal Welfare Commissioner of the University of Veterinary Medicine Hannover approved the experimental procedure in accordance with the German Animal Welfare law (permit number: DFG CE186/2-1).

### Animals, diet and sample collection

Twelve adult, healthy warmblood horses (body weight [bwt] 534 ± 64.5 kg; age 14 ± 7.5 years) consisting of 10 mares, one stallion and one gelding with no known history of gastrointestinal diseases were used in this study (Table 1). The horses were slaughter animals (killed for reasons other than gastrointestinal-derived diseases) and purchased for this study. The sample size (6 animals per group) was chosen to guarantee, in accordance with the power analysis of the superior DFG project, a statistically assured analysis. Horses were housed in individual boxes (bedding: wood shavings) with ad libitum access to tap water and a salt block consisting of sodium chloride. The horses received meadow hay (1.5 kg/100 kg bwt x d^-1^; Table 2) and crushed oat grains (1.2 g starch/kg bwt x d^-1^; Table 2) in two equal meals per day (9 a.m. and 3 p.m.) during a 3-week adaptation period to cover the energy needs according to the GfE (2014) as quote in advance by Coenen et al. (2011) [25, 26]. Additionally, horses were supplemented either with FOS + inulin (originating from Jerusalem artichoke meal [JAM], LIVEN GmbH, Zossen, Germany; Table 2) with the intention to reach the recommended prebiotic dosage of 0.2 g/kg bwt x d^-1^ [13] or with an equal amount of maize cob meal without grains as placebo (CON; Table 2). To avoid selective feed intake and the aspiration of fine particles, water was added (6% of the final volume) to the concentrated meal (w/w) prior to feeding. The horses were randomly distributed to either the treatment (n = 6; JAM) or the control group (n = 6; CON).

**Table 1:** Population characteristics of the horses.

group	gender	age [years]	body weight [kg]
CON	female	3	395
	male	5	560
	female	21	461
	female	12	528
	female	18	570
	female	23	582
JAM	female	11	500
	male castrated	5	590
	female	19	548
	female	20	624
	female	11	570
	female	24	480

CON = placebo group; JAM = Jerusalem artichoke meal

**Table 2:** The analyzed chemical composition of the feedstuffs and diets.

nutrient		oat	hay	placebo^1^	prebiotic^2^	diet
DM	g/kg	912	944	927	939	940
CA	g/kg DM	29	52	26	136	50
CP	g/kg DM	124	90	36	63	93
AEE	g/kg DM	49	9	8	6	14
CF	g/kg DM	124	349	343	14	318
NDF	g/kg DM	316	651	799	3^3^	605
ADF	g/kg DM	178	391	415	11^3^	361
ADL	g/kg DM	31	47	39	4^3^	44
glucose	g/kg DM	0	30	9	14	26
fructose	g/kg DM	0	39	10	63	34
sucrose	g/kg DM	11	6	16	122	8
fructan	g/kg DM	0	40	14	466	38
starch	g/kg DM	498	0	0	0	65
ME (according to [36])	MJ/kg DM	12.4	6.6	7.5	11.9	7.5

The analyzed proximate nutrients are ADF (acid detergent fiber), ADL (acid detergent lignin), AEE (acid ether extract), CA (crude ash), CF (crude fiber), CP (crude protein), DM (dry matter) and NDF (neutral detergent fiber). The ME (metabolizable energy) was calculated according to ^4^.

^1^maize cob meal without grains.

^2^Jerusalem artichoke meal.

^3^The uncommon carbohydrate composition of Jerusalem artichoke meal causes partly paradoxical results in detergent fiber analysis, which is, however, not worthy of note in the diet calculations because of the small quantities of both the prebiotic supplement in the diet and cell wall carbohydrates in the supplement.

The feed samples were ground to pass through a 1 mm sieve of a standard laboratory sample mill. For starch analysis, the cereal grains were pulverized using a ball mill. The dry matter (DM) content, crude ash (CA), crude protein (CP), acid ether extract (AEE), crude fiber (CF), the Van Soest detergent fibers and sugar were determined according to the official German key book for feed analysis ([27]; methods no. 3.1, 4.1.1, 5.1.1 B, 6.1.1, 6.5.1, 6.5.2, 6.5.3, 7.1.2 and 8.1). The content of nitrogen-free extracts (NFE) was calculated (NFE = OM–CP–AEE − CF). Starch was enzymatically determined by the amyloglucosidase method ([27], method no. 7.2.5). The contents of individual water-soluble carbohydrates (WSC; glucose, fructose, sucrose, fructans) and the degree of polymerization (DP) of FOS and fructopolysaccharides in feedstuffs and digesta were investigated using HPLC as described by [28, 29].

The horses were euthanized at d21 of the feeding period (approximately 1 hour after the morning meal) by a veterinarian using 0.12 mg/kg bwt romifidine as a sedative. A combination of diazepam (0.05 mg/kg bwt) and ketamine (2.2 mg/kg bwt) was used for introducing the anesthesia. The horses were euthanized with pentobarbital (60 mg/kg bwt). Immediately post mortem, luminal digesta were collected from 7 different parts of the GIT (stomach: *pars nonglandularis*, PN; *pars glandularis*, PG; small intestine, SI; caecum, CAE; colon: ventrale, CV; dorsale, CD; transversum, CT) and stored at—20 °C until further analysis for proximate nutrients (e. g., starch, WSC, DP) and the microbial composition.

### DNA extraction

Samples (0.25 g) from the different parts of the GIT were weighed and homogenized using sterile 2.0 ml screw-cap tubes containing 0.5 g of 0.1 mm zirconia beads, 4 glass beads (3 mm), and 1.0 ml lysis buffer (500 mM sodium chloride, 50 mM Tris-hydrochloric acid at pH 8, 50 mM EDTA, 4% SDS and double-distilled water (ddH_2_O)). Samples were homogenized by double bead beating (5.5 ms for 3 x 1 min) at room temperature (RT) using a bead beater (Precellys 24, Bertin Technologies, France). After the homogenization steps, samples were heated at 95 °C for 15 min and centrifuged at 4 °C for 5 min (full speed; 15,000 rpm). After cell lysis, nucleic acids were precipitated by adding 260 μl of 10 M ammonium acetate, isopropanol (1 mL) and 500 μl ethanol (70% v/v). Subsequently, the nucleic acid pellet was dissolved in 200 μl TE buffer (containing 10 mM Tris-hydrochloric acid, 1 mM EDTA and ddH_2_O). Finally, DNA was extracted using the commercial QIAmp DNA stool Mini Kit (Qiagen Ltd., UK) according to the manufacturer`s manual. The DNA content was measured using a NanoDrop 2000 (Thermo Fisher Scientific Inc., USA).

### PCR amplification

Samples were amplified in two steps as described previously [30]. Specifically, the first PCR was used to amplify the V1-V2 variable region of the bacterial 16S ribosomal RNA (rRNA) gene using specific linker primers (forward primer: UniTag 1–27F-DegS: GAGCCGTAGCCAGTCTGCGTTYGATYMTGGCTCAG; reverse primer: UniTag 2-338R–I: GCCGTGACCGTGACATCGGCWGCCTCCCGTAGGAGT, UniTag 2–338R–II: GCCGTGACCGTGACACGGCWGCCACCCGTAGGTGT). For each sample, the PCR master mix with a final volume of 20 μl contained 4 μl 5x HF buffer (Fermentas, Thermo Fisher Scientific Inc., USA), 1 μl forward linker primer (UniTag1-27R-DegS, 10 μM), 1 μl reverse linker primer (UniTag2–338R-I + II, 10 μM), 0.4 μl dNTPs (Roche Nederland B.V.), 0.2 μL Phusion Hot start II DNA polymerase (Finnzymes, Thermo Fisher Scientific Inc., USA; 2 U/μl) and 12.4 μl nuclease free water (Promega Corporation, Madison, Wisconsin, USA). Each reaction tube contained 1 μl of DNA template at 10–20 ng/μl. The PCR was performed under the following conditions: hot start at 98 °C for 30 s, followed by 25 cycles of 98 °C for 10 s, 56 °C for 20 s, 72 °C for 20 s and a final extension at 72 °C for 10 min using a Labcycler (Sensoquest, Göttingen, Germany). The PCR product was analyzed using the Lonza Flash Gel system (Lonza Group Ltd., USA). In the second PCR, specific barcoded primers (see S1 Table) were used. The master mix (final volume 85 μl consisting of 20 μl 5 x HF buffer (Fermentas, Thermo Fisher Scientific Inc., USA), 2 μl dNTPs (Roche Nederland B.V.), 1 μl Phusion Hot start II DNA polymerase (Finnzymes, Thermo Fisher Scientific Inc., USA; 2 U/μl) and 62 μl nuclease free water) was mixed with 5 μl of the PCR product from the first step PCR and run under the following conditions: heating at 98 °C for 30 s followed by 5 cycles of 98 °C for 10 s, 52 °C for 20 s, 72 °C for 20 s and a final extension at 72 °C for 10 min using also a Labcycler (Sensoquest, Göttingen, Germany).

### PCR purification and sequencing

PCR products were purified using a HighPrep PCR paramagnetic beads solution (MagBio Genomics Inc., USA). In brief, the PCR tube was mixed with the paramagnetic bead solution, placed on a magnetic separation device and cleaned up using 70% (v/v) ethanol several times. Finally, the sample was eluted with nuclease-free water, and the DNA content was measured with a NanoDrop 2000 (Thermo Fisher Scientific Inc., USA).

For library preparation, the DNA content of each sample was measured with a fluorometer (Qubit 2.0 fluorometer, Thermo Fisher Scientific Inc., USA) using a dsDNA BR assay kit. Each library consisted of 46 samples and 2 mock communities serving as internal controls (S1 Table). Library pools were prepared to contain equimolar amounts of sample DNA at 100 ng of each sample per library pool. If the final volume of the library was above 100 μl, the library was concentrated to 20 μl using the paramagnetic beads solution as before. DNA content was measured again with the Qubit 2.0 fluorometer and set to 100 ng/μl. If necessary, the library was diluted with nuclease-free water to reach a DNA concentration of 100 ng/μl in a final volume of 20 μl. Finally, the samples were sequenced on an Illumina MiSeq sequencer by GATC Biotech AG (Konstanz, Germany).

### Bioinformatic and statistical analysis

The sequencing output data were analyzed using QIIME software [31]. Barcode and primer sequences were removed, and the data were checked to identify and remove chimeras by using the chimera slayer [32]. The resulting sequences were clustered into OTUs at a discrimination level of 97% identity by using the SILVA database version 119 [33]. Each sample was rarefied at a cut-off of 2500 reads, alpha diversity indices (Simpson and Shannon-Wiener), species richness (Menhinick) and beta diversity (Whittaker) were calculated in PAST (version 3.1; [34]), and the results were imported into Microsoft Excel for further analysis. Statistical analyses were conducted using SAS 9.4 (SAS Inc., Cary, NC, USA). The nonparametric Wilcoxon rank-sum test was used to compare the relative abundance of microbial taxa in the different parts of the GIT between the two feeding groups. Diversity indices in the several tracts and the population characteristics of both feeding groups were compared with the t-test implemented in PROC mixed. The principal component analysis was conducted in PAST. Differences in the specific parts of the GIT and between the two feeding groups were tested *via* one-way ANOSIM of ranked Bray-Curtis similarity indices. The unweighted UniFrac distances [35] were calculated in QIIME [31], imported in PAST [34] and analyzed *via* one-way ANOSIM. Furthermore, the Bonferroni correction was applied on the PCoA data for the different regions of the digestive tract. The significance level was set at *P* < 0.05. The sequencing data are available at the European Nucleotide Archive (ENA) under the accession number PRJEB31758.

## Results

### General observations

The feeding group composition was not significantly different between CON and JAM regarding to age and body weight distribution (*P* > 0.05).

All horses accepted the provided feed well and showed no clinical signs of gastrointestinal disturbances throughout the duration of the study. One young mare (age 3 years) belonging to the placebo group showed mucosal lesions alongside the *margo plicatus* in the stomach post mortem.

### Diet components

The chemical analysis of the commercially available JAM meal revealed a much lower content of FOS and inulin than declared (62.7% declared *vs*. 46.6% analysed). Therefore, the intake of the prebiotic active compound achieved was lower than calculated (0.15 *vs*. 0.2 g/kg bwt x d^-1^). Furthermore, the DP was not as high as expected for FOS and inulin (S1 Fig).

The chemical composition of the diet components are presented in Table 2. The placebo contained higher concentrations of fibre (CF: 343 g/kg DM, NDF: 799 g/kg DM, ADF: 415 g/kg DM, ADL: 39 g/kg DM) whereas the prebiotic compound contained higher concentrations of water-soluble carbohydrates (e.g. fructose: 63 g/kg DM, sucrose: 122 g/kg DM, fructan: 466 g/kg DM).

### Digesta composition of water-soluble carbohydrates including starch

The composition of the digesta from the different parts of the GIT regarding WSC, including starch, is presented in S2 Table. No significant differences were observed between the feeding groups or between the different parts of the gastrointestinal tract.

**Fig 1:**
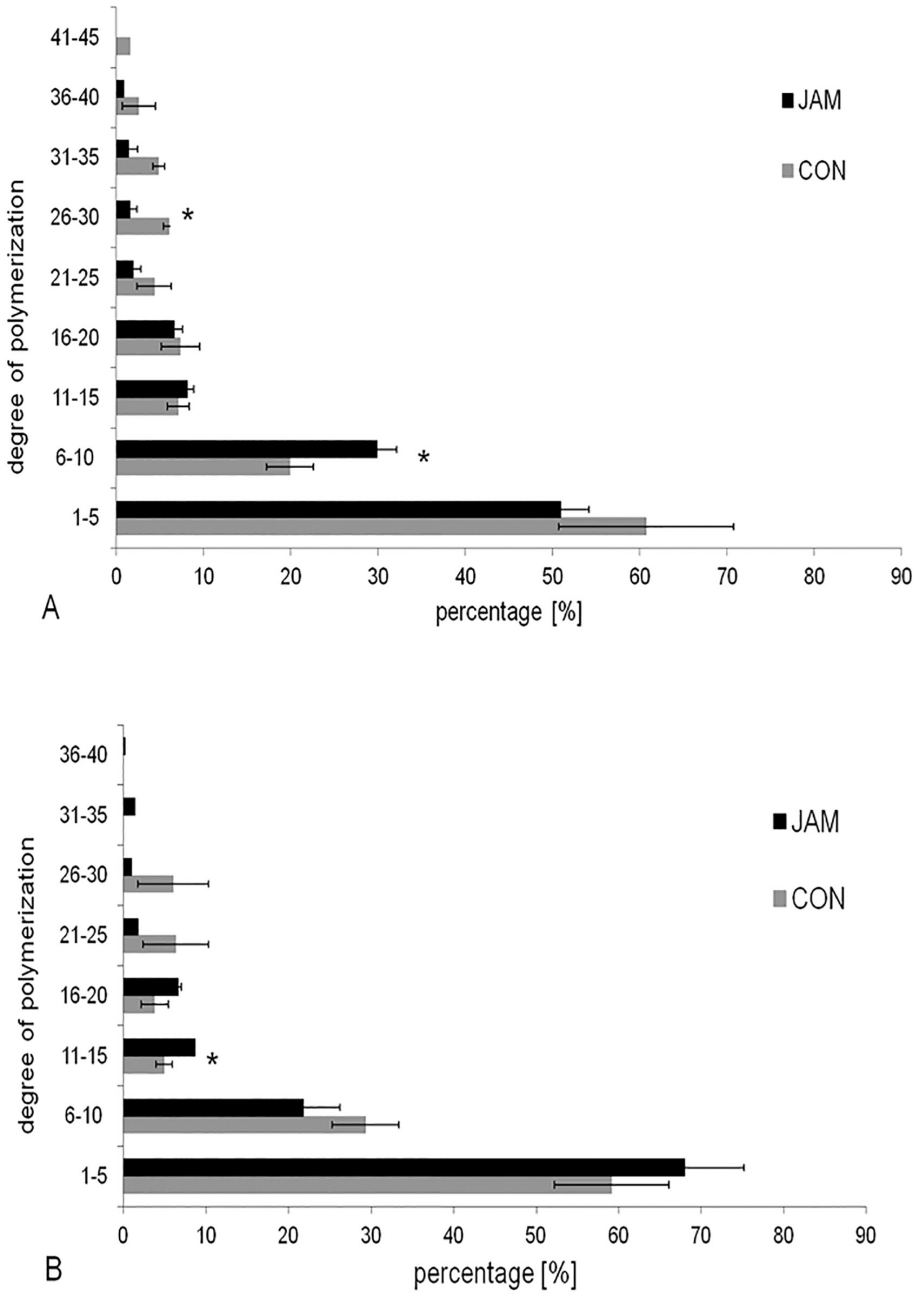
Distribution of the degree of polymerization (%, mean ± SE) in the stomach. The figure A presents the degree of polymerization in the *pars nonglandularis* for the CON (placebo) and JAM (Jerusalem artichoke meal) groups. The asterik (*) indicates significant differences (*P* < 0.05). The figure B presents the degree of polymerization in the *pars glandularis* for the CON (placebo) and JAM (Jerusalem artichoke meal) groups. The asterik (*) indicates significant differences (*P* < 0.05).

The DP of the digesta content in the stomach is presented in Fig 1. The PN contained 61% carbohydrates with a DP of 1–5 units in the treatment group and 20% of molecules had a DP of 6–10 in the CON group. Longer chains were presented by < 10% of the entire composition. In the PG, the quantity of the chains with a DP of 1–5 decreased slightly to 59% and the chains with a DP of 6–10 increased to 29%. Equally, in the JAM group, the largest amount of chains contained 1–5, as well as 6–10, molecules in both parts of the stomach. In contrast to the CON group, the percentage of the shorter chains (DP 1–5) in the stomach content of the JAM group increased from 51% (PN) to 68% (PG).

### 16S rRNA gene MiSeq sequencing data

The sequencing of the samples generated 2,933.868 reads with a mean total number of bases sequenced per sample of 27,419.327 (min–max: 249–204,888). During downstream analysis in the QIIME pipeline, eight samples with fewer than 2,500 reads per sample were excluded (see S1 and S3 Tables). Therefore, 2,920.961 reads were retained for further analysis. The plateau of the rarefraction curve (S2 Fig) indicated an adequate sub-sampling size.

### Diversity

The calculated diversity indices are presented in Tables 3 and 4. The alpha diversity (Simpson and Shannon-Wiener) indicated a higher diversity in all parts of the GIT for the JAM group in comparison to the CON group (*P* < 0.05). Particularly, in the large intestine (CAE, CV, CD and CT), the mean diversity (Simpson 1-D) was higher in the JAM group than in the CON group (*P* < 0.05). Furthermore, the Simpson evenness index showed a more evenly distributed microbial species in the JAM group compared to the CON group in the large and small intestine (Table 3, *P* < 0.05) but not in the stomach (*P* > 0.05). The species richness (Menhinick) was higher in each part of the digestive tract in the JAM group compared to the CON group. The beta diversity is summarized in Table 4 (for detailed information, see S4 Table). Both feeding groups showed nearly identical low similarity between the digestive compartments. In the JAM group, the species similarity between the PN and CAE/CT as well as CAE and CT were lower compared to the CON group.

**Table 3:** LSmeans (± SE) of the calculated diversity indices (Simpson and Shannon-Wiener), Simpson´s evenness and species richness (Menhinick) of the two feeding groups in the different parts of the gastrointestinal tract.

item		PN	PG	SI	CAE	CV	CD	CT
Simpson 1-D	CON	0.495 ± 0.005	0.487 ± 0.017	0.514 ± 0.017	0.533 ± 0.003	0.535 ± 0.008	0.523 ± 0.009	0.535 ± 0.009
	JAM	0.589 ± 0.005	0.578 ± 0.016	0.646 ± 0.019	0.722 ± 0.003	0.707 ± 0.008	0.719 ± 0.009	0.713 ± 0.009
	p value	< 0.001	0.0039	0.0006	< 0.001	< 0.001	< 0.001	< 0.001
Shannon-Wiener	CON	0.969 ± 0.022	0.934 ± 0.062	1.125 ± 0.092	1.434 ± 0.043	1.430 ± 0.067	1.328 ± 0.084	1.449 ± 0.076
	JAM	1.089 ± 0.022	1.055 ± 0.057	1.355 ± 0.101	2.012 ± 0.055	1.868 ± 0.067	1.975 ± 0.084	1.941 ± 0.076
	p value	0.0052	0.1822	0.1274	0.0002	0.0010	0.0003	0.0010
Simpsons evenness	CON	0.290 ± 0.045	0.285 ± 0.024	0.278 ± 0.021	0.164 ± 0.011	0.177 ± 0.009	0.176 ± 0.009	0.185 ± 0.010
	JAM	0.403 ± 0.045	0.334 ± 0.022	0.400 ± 0.023	0.320 ± 0.014	0.299 ± 0.009	0.282 ± 0.009	0.290 ± 0.010
	p value	0.1096	0.1671	0.0040	0.0001	< 0.001	< 0.001	< 0.001
Menhinick	CON	5.539 ± 0.594	5.201 ± 0.258	6.437 ± 0.661	14.926 ± 0.924	13.767 ± 0.816	12.900 ± 1.542	13.493 ± 0.603
	JAM	5.667 ± 0.866	6.253 ± 0.430	7.083 ± 0.585	16.797 ± 1.514	15.695 ± 1.352	18.238 ± 0.924	17.333 ± 1.282
	p value	0.9055	0.0780	0.7825	0.3015	0.2500	0.0141	0.0219

The following parts of the GIT were examined: CAE (caecum), CD (colon dorsale), CT (colon transversum), CV (colon ventrale), PG (*pars glandularis*) PN (*pars nonglandularis*) and SI (small intestine) in the CON (placebo group fed with maize cob meal without grains) and in the JAM group (Jerusalem artichoke meal). The p value refers to the difference of means between both feeding groups within the calculated indices.

**Table 4:** LSmeans (± SE) of the calculated beta diversity index (Whittaker) of the two feeding groups in relation to the different regions of the gastrointestinal tract.

compared GIT regions	CON	JAM	p value
PN—PG	0.273 ± 0.073	0.302 ± 0.049	0.7427
PN—SI	0.469 ± 0.038	0.387 ± 0.093	0.4381
PN—CAE	0.626 ± 0.058	0.661 ± 0.075	0.7247
PN—CV	0.616 ± 0.066	0.618 ± 0.042	0.9845
PN—CD	0.568 ± 0.081	0.681 ± 0.062	0.2998
PN—CT	0.620 ± 0.101	0.698 ± 0.043	0.4927
PG—SI	0.425 ± 0.034	0.454 ± 0.069	0.7077
PG—CAE	0.677 ± 0.031	0.670 ± 0.044	0.8915
PG—CV	0.674 ± 0.018	0.641 ± 0.021	0.2628
PG—CD	0.644 ± 0.056	0.708 ± 0.038	0.3565
PG—CT	0.720 ± 0.017	0.711 ± 0.039	0.8442
SI—CAE	0.667 ± 0.019	0.653 ± 0.022	0.6494
SI—CV	0.671 ± 0.012	0.596 ± 0.047	0.1275
SI—CD	0.672 ± 0.038	0.674 ± 0.036	0.9817
SI—CT	0.688 ± 0.023	0.695 ± 0.039	0.8756
CAE—CV	0.195 ± 0.047	0.238 ± 0.055	0.5863
CAE—CD	0.218 ± 0.047	0.233 ± 0.054	0.8374
CAE—CT	0.324 ± 0.019	0.313 ± 0.027	0.7361
CV—CD	0.274 ± 0.055	0.237 ± 0.027	0.5542
CV—CT	0.274 ± 0.028	0.305 ± 0.025	0.4339
CD—CT	0.283 ± 0.050	0.234 ± 0.038	0.4547

The following GIT regions were examined: CAE (caecum), CD (colon dorsale), CT (colon transversum), CV (colon ventrale), PG (*pars glandularis*) PN (*pars nonglandularis*) and SI (small intestine) in the CON (placebo group fed with maize cob meal without grains) and in the JAM group (Jerusalem artichoke meal). Each part of the GIT was compared with the other parts of the GIT. The p value refers to the differences of means within the individual parts of the gut.

### Microbial composition in relation to the different intestinal sections examined

**Fig 2:**
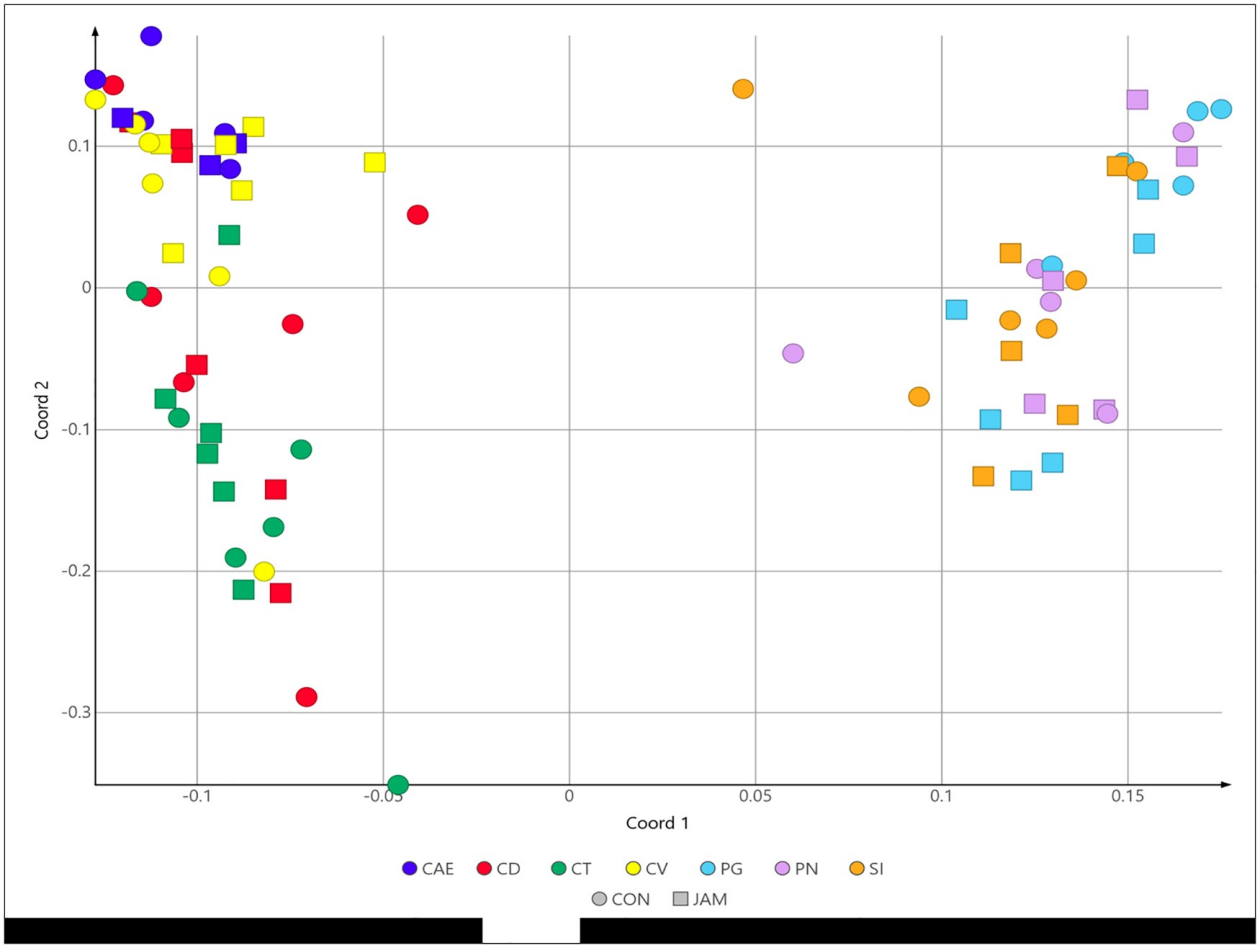
Principal component analysis (PCoA) of the microbial composition in the digestive tract. Unweighted UniFrac distance measures were used for the PCoA. The following parts of the GIT were examined: CAE (caecum), CD (colon dorsale), CT (colon transversum), CV (colon ventrale), PG (*pars glandularis*) PN (*pars nonglandularis*) and SI (small intestine) in the CON group (placebo group fed with maize cob meal without grains) and in the JAM group (Jerusalem artichoke meal).

Fig 2 illustrates the within-compartment similarity along the equine gastrointestinal tract. Axis 1 describes 46.6% of the total variation and axis two 9.3% of the variation. The principal component analysis indicates a differentiation of the specific microbial composition in the distinct parts of the GIT (P < 0.05; Table 5). Nonetheless, the analysis revealed no obvious separation of the microbial community in the JAM group in comparison to the CON group (P > 0.05).

**Table 5:** Pairwise ANOSIM of Bray-Curtis similarity indices between the different regions of the GIT.

	PG	CV	CD	CT	PN	SI	CAE
PG	-	1.0000 0.0805	0.1764	**0.0021**	**0.0021**	**0.0021**	**0.0021**
CV	0.0805	-	0.1260	**0.0021**	**0.0021**	**0.0021**	**0.0021**
CD	**0.0084**	**0.0060**	-	**0.0021**	**0.0021**	**0.0021**	**0.0021**
CT	**0.0001**	**0.0001**	**0.0001**	-	1.0000	1.0000	**0.0336**
PN	**0.0001**	**0.0001**	**0.0001**	0.2709	-	1.0000	0.4956
SI	**0.0001**	**0.0001**	**0.0001**	0.6151	0.9111	-	1.0000
CAE	**0.0001**	**0.0001**	**0.0001**	**0.0016**	**0.0236**	0.1190	-

The results indicate the one-way ANOSIM testing of ranked Bray-Curtis similarity indices between the different regions of the GIT: CAE (caecum), CD (colon dorsale), CT (colon transversum), CV (colon ventrale), PG (*pars glandularis*) PN (*pars nonglandularis*) and SI (small intestine). The p values are shown in the upper left and the Bonferroni corrected p values of the pairwise comparisons are shown in the upper right. Differences written in bold were significant.

**Fig 3:**
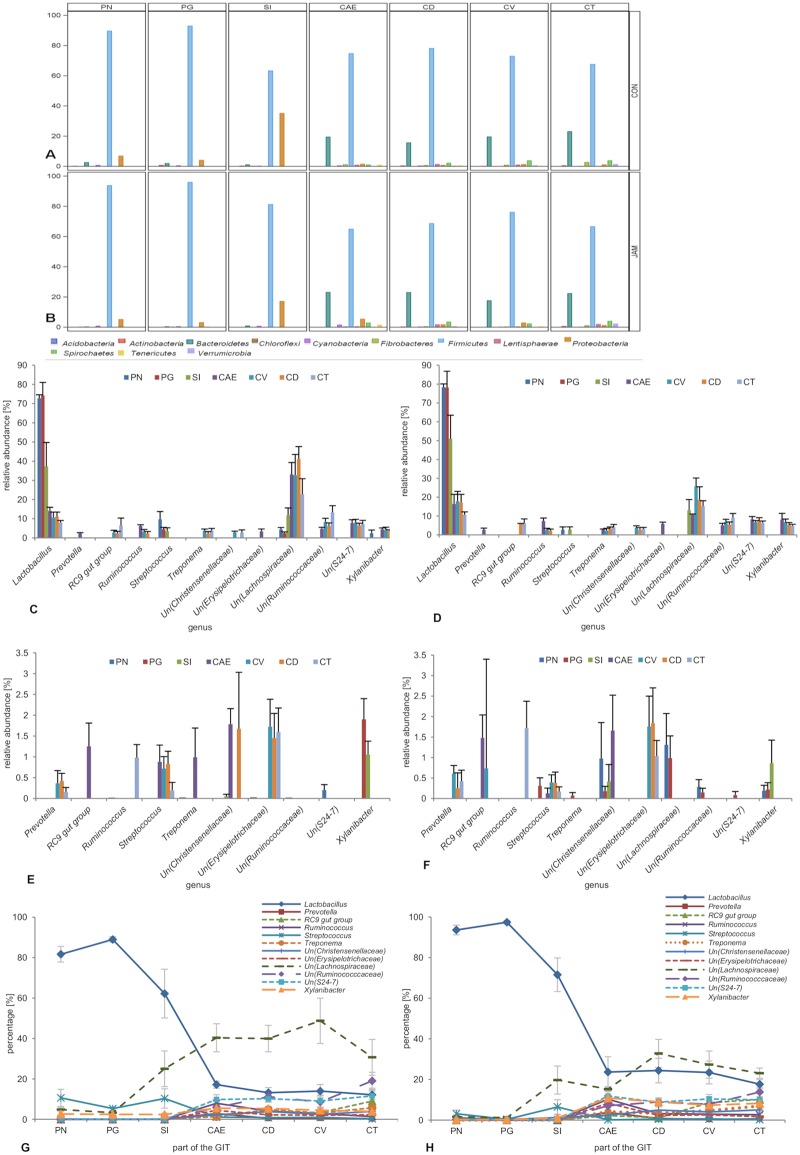
Mean relative abundance (% ± SE) of the different phyla (A–B), the most abundant genera (mean relative abundance ≥ 2%, C-D), rare genera (mean relative abundance < 2%, E-F) and the distribution of the 12 most abundant genera (G-H) along the equine gastrointestinal tract. The following parts of the digestive tract were examined: CAE (caecum), CD (colon dorsale), CT (colon transversum), CV (colon ventrale), PG (*pars glandularis*), PN (*pars nonglandularis*) and SI (small intestine). Figs 3A, 3C, 3E and 3G represent the CON group (placebo). Figs 3B, 3D, 3F and 3H illustrate the JAM group (Jerusalem artichoke meal). Both feeding groups included Un (an unknown member of a specific family).

In the foregut (including the PN, PG and SI), the supplemental feeding of JAM resulted in a generally higher relative abundance of the phylum *Firmicutes* (Fig 3A and 3B, no significant difference) and a lower abundance of *Bacteroidetes* (PG: *P* < 0.05) and *Proteobacteria* (no significant difference) compared to supplemental feeding of the placebo (CON). In the large intestine, the addition of JAM increased the relative abundance of *Bacteroidetes* (no significant difference), *Proteobacteria* (no significant difference) and *Spirochaetes* (no significant difference) in the CAE, as well as in the CD, but it reduced the abundance of *Firmicutes* (no significant difference) in both GIT regions. Furthermore, the relative abundance of the phylum *Proteobacteria* increased in the CV of the JAM group, but almost no variation was observed in the CT in comparison to the CON group (no significant difference).

The sequencing data revealed the presence of 100 different taxa at the genus level in the whole GIT (S5 Table). For statistical analysis, genera with a frequency of ≥ 2% for at least one treatment (CON or JAM) and one intestinal part were included. Hence, 12 different genera remained for statistical comparison (Fig 3C–3H). The most abundant genus in the stomach (both sections) and small intestine was *Lactobacillus* (no significant difference between feeding groups, Fig 3C and 3D). The second most abundant genus was *Streptococcus* in the CON group (PN and PG; CON vs. JAM: P < 0.05) but only partly in the JAM group (here: PN). In the SI, an unclassified member of the family *Lachnospiraceae* (*Un (Lachnospiraceae)*) was the second most abundant genus in both groups. In the PN, the unclassified member of the family *Lachnospiraceae* (*Un (Lachnospiraceae)*) was in the CON group compared to the JAM group (P < 0.05). *Lactobacillus* was abundant in all parts of the large intestine but not the dominant genus. The most abundant genus in the large intestine was represented by the same uncultured species from the family *Lachnospiraceae* (*Un (Lachnospiraceae)*), as in the foregut, with higher abundance in the CON group compared to the JAM group (significant difference only in the CAE, P < 0.05). Furthermore, the genus *Xylanibacter* was found at higher relative abundance in the JAM group than the CON group (only in the PG: *P* < 0.05).

Rare bacterial genera (mean relative abundance ≤ 2%) were primarily present in the large intestine of the CON group (Fig 3E), whereas in the JAM group, these genera were also present in the foregut (Fig 3F). The hindgut in the CON and JAM groups was dominated by an unknown member of the family *Christensenellaceae* and *Erysipelotrichaceae*. The foregut of the JAM group was additionally dominated by an unknown member of the family *Lachnospiraceae*.

Fig 3G and 3H show the distribution of the most abundant genera along the equine gastrointestinal tract. The two feeding groups differed in the mean relative abundance of the genus *Lactobacillus*, which was higher in the JAM group, especially in the hindgut. Moreover, the mean relative abundance of an unknown member from the family *Lachnospiraceae* increased to a higher percentage in the CON-fed group compared to the JAM-fed group.

## Discussion

The horses ingested the provided feed very well and showed no visible clinical signs of gastrointestinal disturbances or other diseases. Therefore, the calculated and ingested amounts of the prebiotic active compounds were likely within a healthy range. Surprisingly, one young mare of the CON group showed mucosal lesions alongside the *margo plicatus* (*pars nonglandularis*) in the stomach post mortem. According to the classification of the ECEIM [37], the ulcers are primarily classified in the EGUS grade 3 with some individual lesions grade 2. Because the horses used in this study differed in a number of characteristics, e.g., age, body weight, feeding and housing conditions prior to the study, the young mare might have developed the mucosal lesions before starting the experiment.

The microbial composition in the equine GIT is prone to disturbances, primarily as a result of dietary changes. The horses in the present study had a starch intake of 1.2 g/kg bwt x d^-1^ *via* the feed concentrate with a hay to concentrate ratio of 87:13 (as fed basis), which was therefore clearly a forage-based diet. The starch supply was only slightly above what is recommended to be safe [25, 38; ≤ 1 g starch/kg bwt x d^-1^] and should not have caused an overwhelming microbial fermentation and production of organic acids in the stomach.

A total of 0.2 g/kg bwt x d^-1^ of FOS + inulin for horses is recommended as an effective prebiotic dose in horses [13], meaning to achieve health-promoting effects *via* the GIT. Analyzing the prebiotic Jerusalem artichoke meal after completion of the current study revealed, however, a lower amount of the prebiotic active compound than declared (46.6% analyzed *vs*. 62.7% declared). Therefore, the horses ingested only 0.15 g/bwt x d^-1^ FOS + inulin instead of the intended 0.2 g/kg bwt x d^-1^. Furthermore, the effect of a prebiotic on the GIT also depends on its chemical composition, especially the DP of oligo- and polymeric carbohydrates [39]. The Jerusalem artichoke meal used in the study showed a lower percentage of higher DP molecules (23.3% for chains from 16–20 monomers and 21.1% for chains from 11–15 monomers) than described in the literature (inulin-type fructans: DP 2–60 [12]). As a consequence, the amount of the prebiotic active compound reaching the large intestine was likely lower than intended, and therefore, the effect on the microbial community might have been reduced. Recent literature confirmed the assumption, that the natural prebiotic might in part already be degraded in the stomach [40].

Glatter et al. (2016), who used the same animal material as in the current study, reported a higher impact of Jerusalem artichoke meal on microbial fermentation in the foregut, especially the stomach, in comparison to the large intestine [21]. Mainly in the digesta of the nonglandular part of the stomach, the concentration of SCFAs (primarily *n*-butyric acid) was twice as high after feeding of Jerusalem artichoke meal *vs*. control feeding. This finding is in accordance with other studies that also described the fermentation of inulin and/or grass fructans starting in the lower gut [20, 40]. The degradation might possibly be started by acid hydrolysis in the stomach [23, 24]. Furthermore, the WSC content in the large intestine was zero in the current study, indicating a largely complete degradation in the foregut. The high amount of short molecules (DP ≤ 10), especially in the stomach and the immediate shortening of carbohydrate chains in this organ supports the assumption that the stomach is a fructan-degrading organ most likely by the autochthonous microbiota [21]. This hypothesis is supported by the results of the current study because the feeding of the Jerusalem artichoke meal resulted in an increased relative abundance of the specific microbial species. The phylum *Firmicutes*, and especially the genus *Lactobacillus* spp. was higher primarily in the stomach in the JAM group compared to the CON group. In accordance with the literature data, *Lactobacillus* was identified as one of the dominant genera in the horses´ stomachs [4, 5, 7, 41, 42, 43]. In addition, we observed a decrease in the relative abundance of *Streptococcus* spp. after feeding JAM, which is also one of the dominant genera in the equine stomach [5, 7, 43]. Perkins et al. (2012) showed that both genera form a thin layer at the gastric mucosa [5]. Consequently, a shift in the microbial composition towards one genus might be crucial for stomach health. In vitro studies confirm the assumption that higher microbial activity with an increasing amount of fermentation products (SCFAs, *n*-butyric acid in particular) in the stomach might have harmful effects on the gastric mucosa [44].

In accordance to previously reported results [7], our results showed that the most abundant phylum in the small intestinal digesta was *Firmicutes* followed by *Proteobacteria*. This was irrespective of the feeding group. In the current study, the digesta of the three different parts of the equine small intestine (duodenum, jejunum and ileum) was mixed and further used as an aggregate sample. Therefore, the results from the small intestine are limited to the mixed digesta whereas other studies have investigated the specific parts separately [7]. Previous studies have indicated that the concentration of bacteria, particularly proteolytic bacteria [6], increases along the foregut in the cranial to caudal direction. This might explain why our results were more likely comparable to the duodenum instead of the ileum.

The feeding of prebiotic active compounds aims to stimulate the metabolism of the autochthonous microbiota mainly in the large intestine [12]. In accordance with other studies, we observed that the dominant phylum in the equine large intestine was *Firmicutes* followed by *Bacteroidetes* [43, 45]. Feeding JAM reduced the relative abundance of an unclassified genus from the family *Lachnospiraceae* in all parts of the large intestine compared to feeding the placebo. Furthermore, several members of the family *Lachnospiraceae* mainly produce butyrate as a fermentation product [46, 47], which has, other as in the stomach [44, 48, 49], several health-promoting effects on the intestine, e.g., as a key energy source for the colonocytes, thus stimulating epithelial cell proliferation and improving barrier function [50]. A reduction of this family might has negative consequences for the health of the hindgut. Glatter et al. (2016) reported an increased concentration of *n*-butyric acid exclusively in the CV in response to feeding JAM indicating that the prebiotic effect was less marked than expected [21], with a lower intake of prebiotic substances than intended.

Except for the CAE, this was also the case for an unclassified genus from the family *Ruminococcaceae*. In contrast, the relative abundance of *Ruminococcus* spp. increased in the CAE and CT with the supplementation of JAM to the diet. Dougal et al. (2013) described a high abundance of the family *Ruminococcaceae* (in descending order) in the feces, CV and CAE [45]. In the current study, no effect was observed on the relative abundance of fibrolytic *Ruminococcaceae* in the CV. *Lachnospiraceae* and *Ruminococcaceae* have a greater relative abundance (in the feces) in healthy horses compared to colic patients [51]. Therefore, a reduction of members from these families might have negative impacts concerning the health of the hindgut.

The sequencing data revealed a high number of genera, which were distributed in the GIT at low relative abundance. Proudman et al. (2015) indicated that these were likely plant- or soil-attached bacteria and therefore belonged to the transient microflora of the GIT [52]. Whether or not and to what extent these bacteria could contribute to metabolism in the digestive tract is currently not known. Furthermore, the recent study used DNA to determine the microbial composition in the different parts of the GIT but this approach is not able to make conclusions about the metabolic activity. The results are limited and do not distinguish between alive or dead microbial species in the GIT which has to be taken into consideration.

Moreover, an increase in bacterial diversity following prebiotic supply, as reported in the recent study, might lead to a more stable microbial community that is less sensitive to biotic/abiotic stressors. The calculated diversity indices (Simpson and Shannon-Wiener) varied between the feeding groups. In all parts of the gastrointestinal tract, the diversity was elevated with the supplementation of JAM to the diet. Furthermore, the species richness was higher in the JAM group than the CON group, which indicates that the different GIT sections might have reduced susceptibility to pathogens and increased microbial community stability [53]. Considering the beta diversity, the different parts of the GIT are self-containing habitats but are not unaffected by each other because of their physiological and anatomical functions. Therefore, the calculated low levels of similarity between the different parts are not unexpected. Moreover, the feeding of JAM resulted in a higher beta diversity concerning in the PN than CAE and CT. This might lead to the assumption that the introduction of a prebiotic into the equine digestive tract (more precisely the microbial community) resulted in a more diverse microbiota at the beginning of the GIT (here: PN) compared to the hindgut. In general, higher diversity in the microbial communities is beneficial for the stability of the ecological system [53, 54] however, it has been shown that microbial diversity declines with age [46] but this was not explicit investigated in this study.

Future studies should include detailed chemical analyses of the content and DP distribution of prebiotic active substances in the prebiotic supplement in question, which is particularly important when no purified FOS/inulin preparations are used. To prevent the breakdown of these substances in the foregut, galenic treatment is required so that the release of the prebiotic active substances occurs first in the large intestine. Because of the expected higher prebiotic load of the large intestine, if such stomach protected prebiotics are used, both the effective and safe dose need a reappraisal based on experimental results.

Finally, future studies should include the application of prebiotics under different biotic and/or abiotic stressors as well as different breeds and ages of horses to better describe the impact of the prebiotics under different physiological conditions. Further considerations might include investigating the different mucosal and luminal microbiota compositions, which might have diverse metabolic functions: however, collecting such samples is invasive and often limited due to ethical reasons. Nevertheless, such studies would be of great advantage, as the results obtained from research projects that use feces provide very little information on the microbiota composition and function in the foregut.

## Conclusion

Despite the large intestine being the declared target for prebiotic interventions, the results of this study show a clear effect in the foregut. The supplementation of Jerusalem artichoke meal containing prebiotic fructooligosaccharides and inulin increased the relative abundance of the dominant genus *Lactobacillus* and decreased the relative abundance of *Streptococcus* to a marked extent in the stomach. This alteration might cause a harmful impact on the stomach by increased bacterial metabolism (SCFA production) and possibly a decreased pH value. Recent literature data indicate, that stomach mucosal lesions might also occur without a markedly pH decrease. Feeding Jerusalem artichoke meal nevertheless increases the bacterial diversity in all parts of the digestive tract, which might be beneficial for the stability of the gastrointestinal microbial community.

## Supporting information

S1 Fig Percentage (%) of the degree of polymerization in the dietary components. (TIF)

S2 Fig Rarefraction curve represents adequate number of species after sub sampling of 2,500 reads per sample. The dashed line indicate the CON (placebo) feeding group and the continuous line indicate the JAM (Jerusalem artichoke meal) feeding group separated for the different parts of the GIT: CAE (caecum), CD (colon dorsale), CT (colon transversum), CV (colon ventrale), PG = (*pars glandularis*), PN (*pars nonglandularis*) and SI (small intestine).
(TIF)

S1 Table Library composition including individual primers for each sample and two mock communities. (XLSX)

S2 Table Water-soluble carbohydrate and starch content in the chyme of the different regions of the gastrointestinal tract (GIT). The water soluble carbohydrates and starch content were analyzed in the CON (placebo) group and the JAM (Jerusalem artichoke meal) group. Values are presented on a DM (dry matter) basis.
(XLSX)

S3 Table Base pairs per sample. (XLSX)

S4 Table Beta diversity in the different regions of the digestive tract and in relation to the feeding groups. The following regions of the digestive tract were compared: CAE (caecum), CD (colon dorsale), CT (colon transversum), CV (colon ventrale), PG = (*pars glandularis*), PN (*pars nonglandularis*) and SI (small intestine) for the CON group (placebo) and the JAM group (Jerusalem artichoke meal).
(XLSX)

S5 Table Relative abundance (in %) at the genus level for the individual horses in the JAM and CON groups. The relative abundances per animal are presented for the CON (placebo) group and the JAM (Jerusalem artichoke meal) group. Both groups contained genera with Un (unknown) members or IS (incertae sedis) species.
(XLSX)
