# Supplementary figures and images for: Modification of the equine gastrointestinal microbiota by Jerusalem artichoke meal supplementation
Source: PLoS One. 2019 Aug 8;14(8):e0220553. doi: 10.1371/journal.pone.0220553 (PMC6687111; doi:10.1371/journal.pone.0220553)

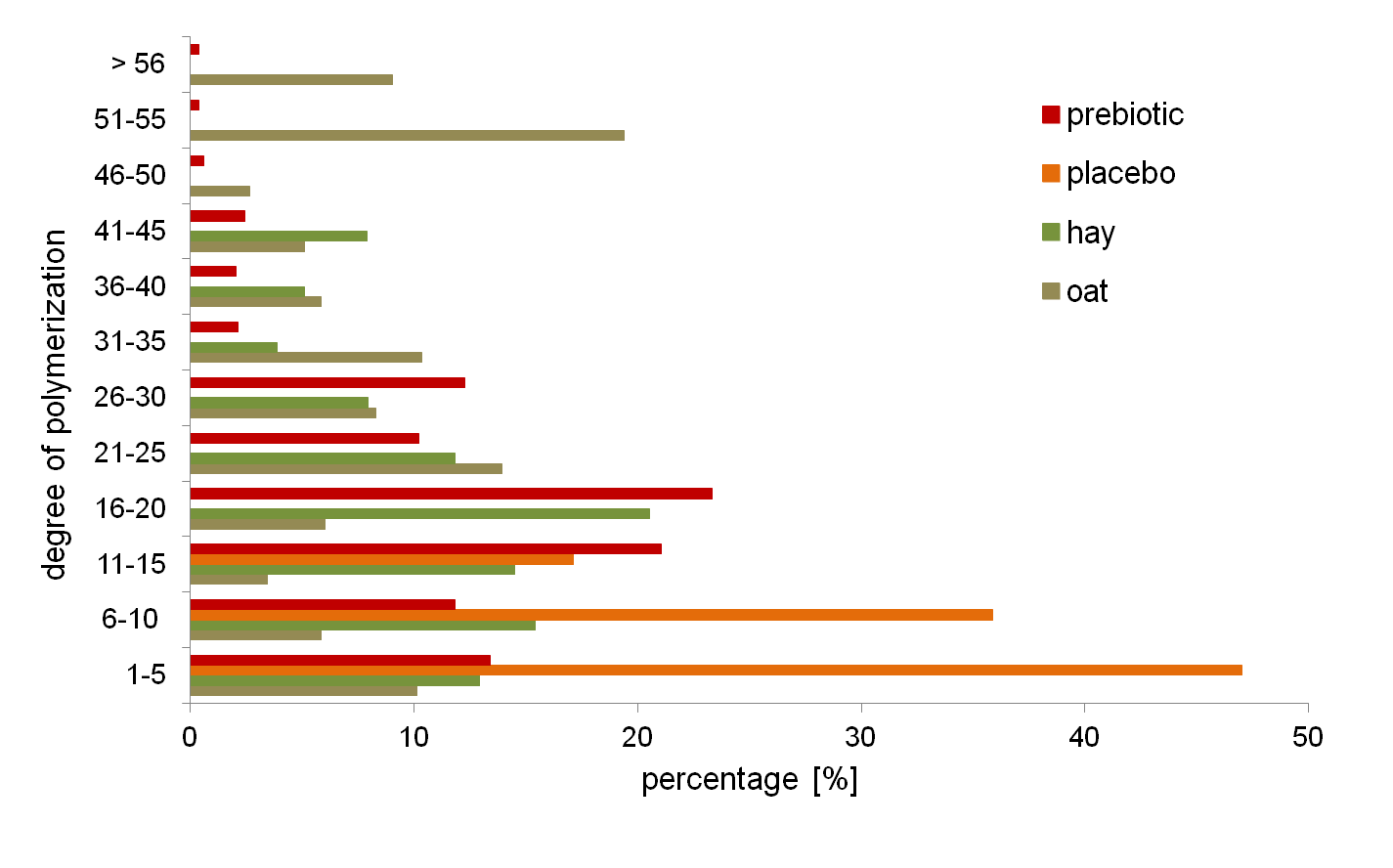

Supplement: S1 Fig — (TIF) [file pone.0220553.s001.tif]

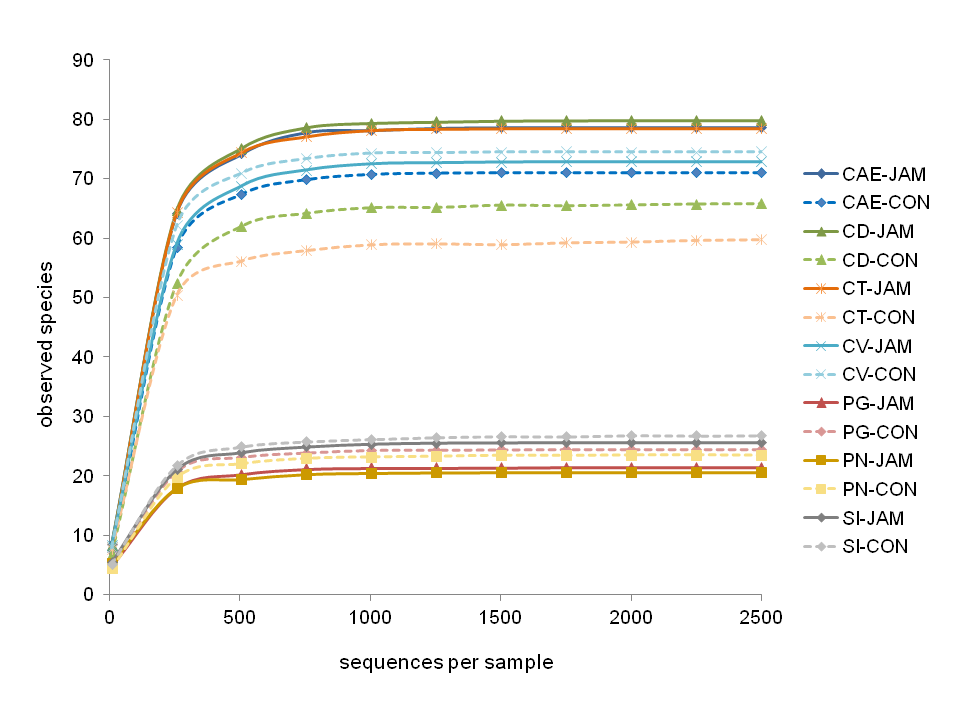

Supplement: S2 Fig — The dashed line indicate the CON (placebo) feeding group and the continuous line indicate the JAM (Jerusalem artichoke meal) feeding group separated for the different parts of the GIT: CAE (caecum), CD (colon dorsale), CT (colon transversum), CV (colon ventrale), PG = (pars glandularis), PN (pars nonglandularis) and SI (small intestine). (TIF) [file pone.0220553.s002.tif]
